# Supplementary material for: Single-cell methylation analysis of brain tissue prioritizes mutations that alter transcription
Source: Cell Genom. 2023 Dec 4;3(12):100454. doi: 10.1016/j.xgen.2023.100454 (PMC10726494; doi:10.1016/j.xgen.2023.100454)
Supplement: Document S1. Figures S1–S6 and Tables S1–S6 [file mmc1.pdf]

Cell Genomics, Volume *3*

Supplemental information

Single-cell methylation analysis of brain tissue prioritizes mutations that alter transcription

Jonathan Flint, Matthew G. Heffel, Zeyuan Chen, Joel Mefford, Emilie Marcus, Patrick B. Chen, Jason Ernst, and Chongyuan Luo

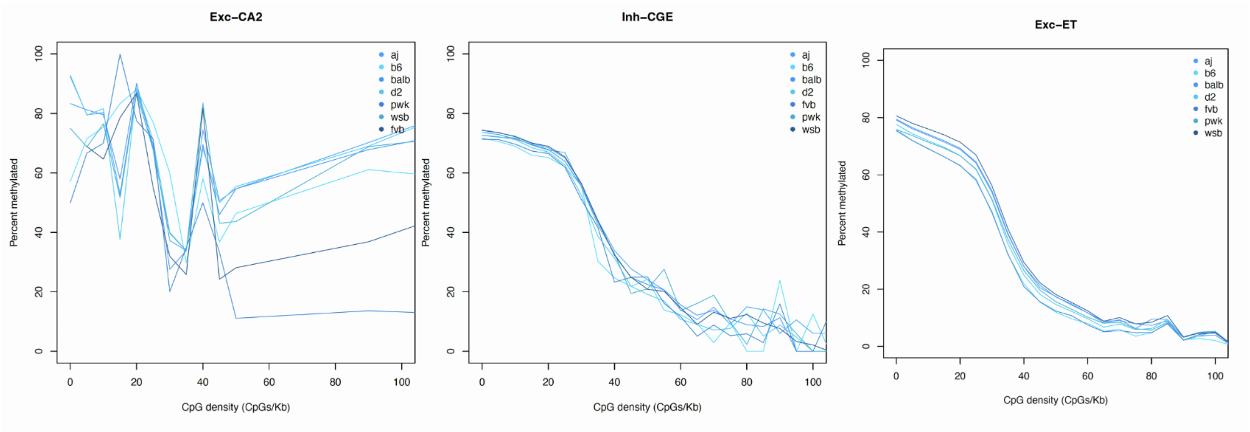

Supplemental Figure S1. Relationship between methylation state and CpG density for three cell types at different genome coverages, related to Figure 2a. The percentage of CpG sites that are methylated is shown on the vertical axis. The horizontal axis is the sequence density of CpG sites (regardless of methylation) per kilobase of genomic DNA. Each blue line represents a different inbred strain where aj = A/J, b6 = C57BL/6J, balb = BALB/cJ, d2 = DBA/2J fvb= FVB/J , pwk = PWK/PhJ and wsb = WSB/EiJ. The cell type names are shown on top of each panel. Mean genome sequence coverages are 7 for Exc-CA2, 16 for Inh-CGE and 36 for Exc-ET. At coverage less than 10, the relationship between CpG density and methylation is invisible due to the large amount of variation in the estimates, but as coverage increases the pattern becomes clear in all celltypes.

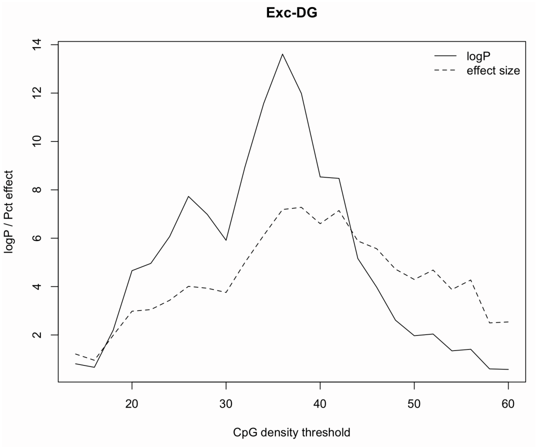

Supplemental Figure S2. Interaction effect between mutation and RNA transcript abundance plotted for different thresholds for CpG density, related to STAR Methods. The horizontal axis shows the threshold at which CpG density was divided into 'high' versus 'low' regions. The vertical axis shows the negative logarithm of the P-value (base 10) for the interaction, and the effect size (multiplied by 100).

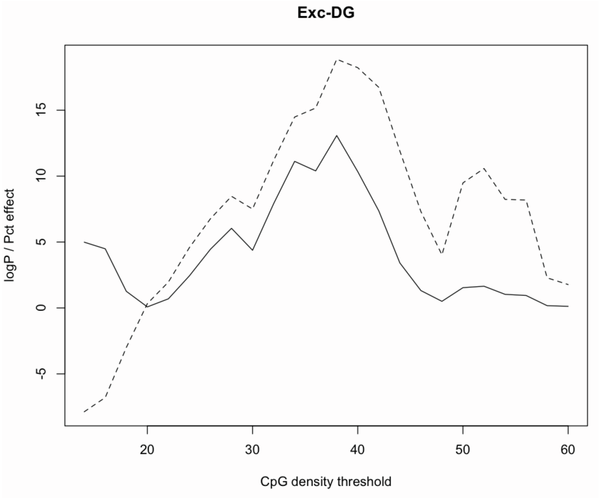

Supplemental Figure S3. Interaction effect between mutation and RNA transcript abundance plotted for different thresholds for CpG density, related to STAR Methods. The horizontal axis shows the threshold at which CpG density was divided into 'high' versus 'low' regions. The vertical axis shows the negative logarithm of the P-value (base 10) for the interaction (dotted line on the graph), and the effect size (multiplied by 100) (solid line on the graph).

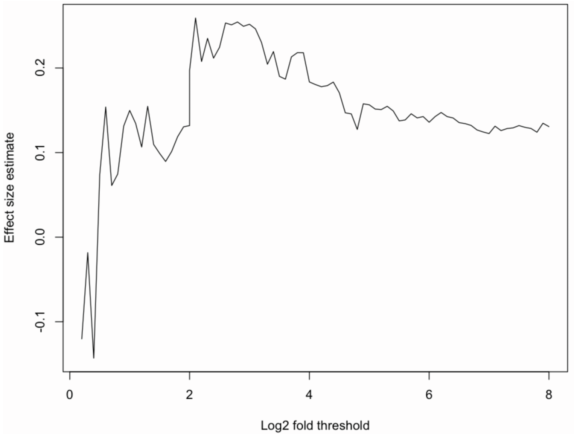

Supplemental Figure S4. Interaction effect sizes between mutation and CpG density at different thresholds for including transcripts, related to STAR Methods. The horizontal axis shows log2 fold thresholds and the vertical axis shows the effect size estimate of the interaction.

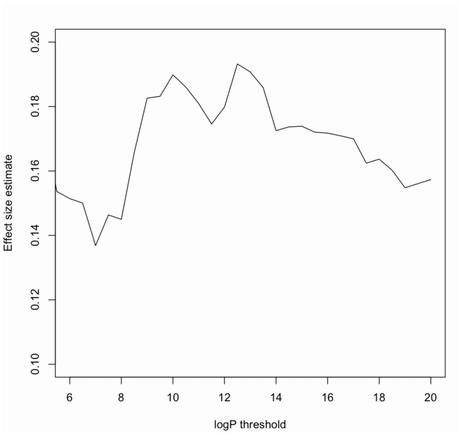

Supplemental Figure S5. Interaction effect size between mutation CpG density for predicting RNA transcript abundance, related to STAR Methods. The x axis shows different -LogP thresholds for including transcripts. The vertical axis shows the effect size estimate of the interaction.

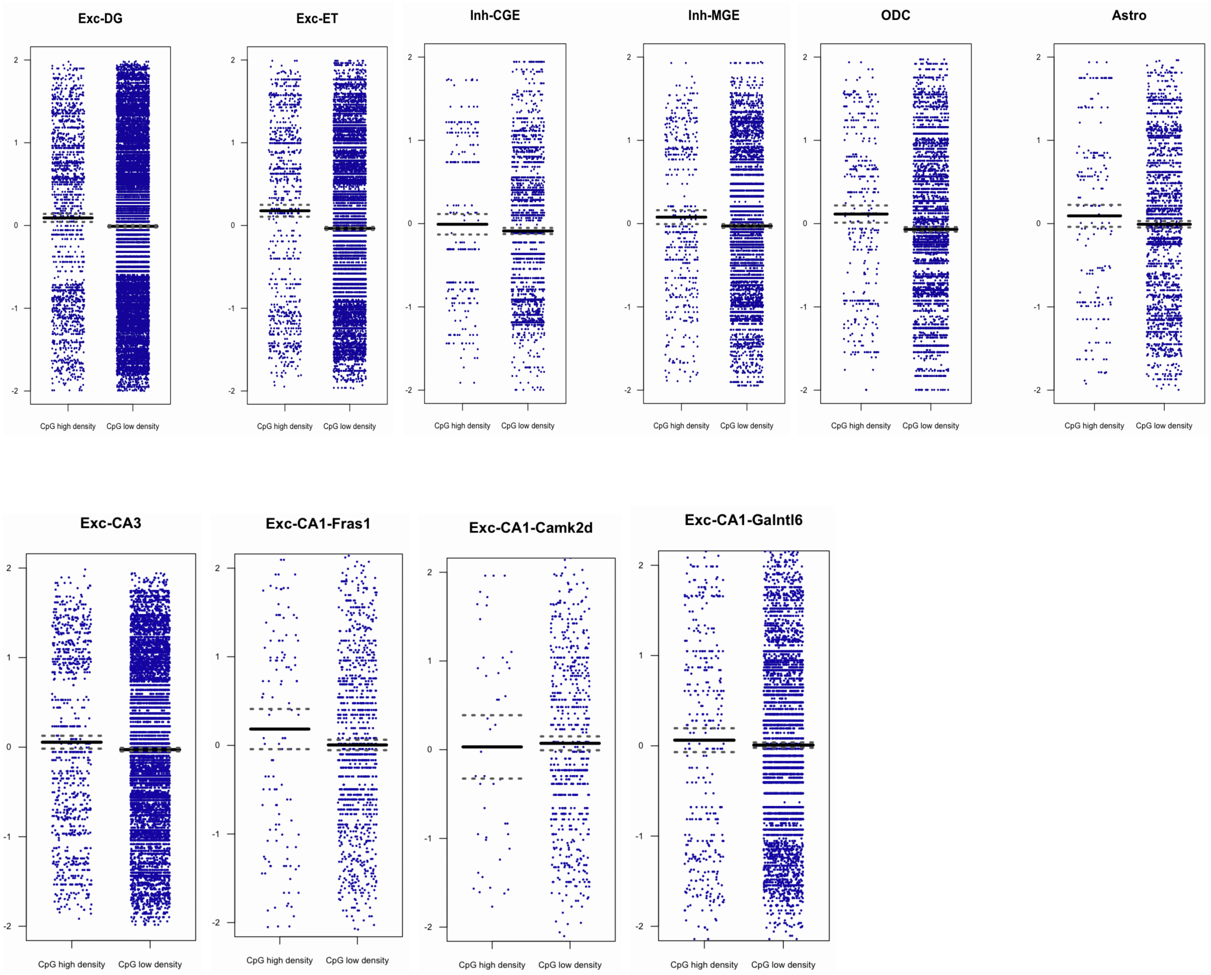

Supplemental Figure S6. Effect of mutations at methylated CpGs on normalized fold change in RNA abundance in regions of low and high CpG density, related to Figure 5. Each plot shows results for a different tissue type. Each dot represents the change in RNA transcript abundance in D2 compared to B6 strains of mice for mutations lying in regions of high CpG density (> 40 CpG/Kb) and in regions of low CpG density (<40 CpG/Kb). The horizontal bars indicate the mean change in RNA transcript, with upper and lower 95% confidence intervals shown as dotted lines. The names of cell types are given at the head of each plot. The first is the Exc-DG cell type reported in the main text. The second is an excitatory cell type, Exc-ET which contains only 10% of the sites used for the Exc-DG set but still demonstrates that mutations in high CpG sequence density regions increase the RNA fold change compared to mutations in low sequence density regions.

Cell type	Mean coverage	Cell type	Mean coverage
Exc-CA2	7.28	MGC	12.81
Exc-Fstl4-Grm3	7.46	Exc-CA1-Lingo2	13.50
EC	7.46	Inh-CGE	16.44
Exc-CA3-Kcnh5	7.53	Exc-Kcnh5-Foxp1	17.16
VLMC	7.56	ODC	20.81
Exc-Gfra1	8.76	Inh-MGE	24.22
Exc-GM45686	9.49	Exc-CA3	25.08
Exc-Kcnh5-Cables1	10.26	Exc-ET	35.85
Astro	11.23	Exc-DG	56.22
OPC	11.80		

Supplemental Table S1. DNA sequence coverage information for each cell type for the single nucleus methylation analyses. Related to Figure 1.

Feature	Df	Sum sq	Mean sq	F-value	P-value
States	16	4864	304.03	398.87	< 2.20E-16
Density	1	2578	2578.44	3382.73	< 2.20E-16
Mutation	1	119	118.51	155.48	< 2.20E-16
Density:Mut.	1	22	21.88	28.71	8.41E-08
Residuals	7215541	5499937	0.76		

Table S2. Results from a linear model that tests the prediction of chromatin states, CpG density, mutation and the interaction between mutation and density on the log2fold change in transcript abundance, related to Figure 5 and STAR Methods. Df is degrees of freedom, Sum sq and Mean sq, the sum and mean squares. The interaction is shown as "Density:Mut" and is significant, consistent with the results shown in the main text.

Fold threshold	Total sites	Interaction effect	Interaction p-value	No. of mutations	No. of mutations in high density
1.9	1,279,002	0.152	3.24E-08	19,242	1,446
1.8	1,267,991	0.133	1.79E-06	19,039	1,413
1.7	1,258,155	0.114	4.58E-05	18,874	1,402
1.6	1,247,122	0.102	2.94E-04	18,537	1,371
1.5	1,221,391	0.112	1.11E-04	17,804	1,313
1.4	1,204,120	0.136	3.34E-06	17,370	1,289
1.3	1,194,103	0.169	1.06E-08	17,127	1,255
1.2	1,169,735	0.118	8.77E-05	16,679	1,202
1.1	1,151,405	0.153	5.39E-07	16,451	1,168
1.05	1,138,464	0.143	3.37E-06	16,278	1,141
1.01	1,119,438	0.158	4.04E-07	15,954	1,120

Supplemental Table S3. Impact of log2 fold threshold for including transcripts in an analysis of interaction between mutation and changes in transcript abundance, related to STAR Methods. The table gives details for analyses performed with fold threshold values between 1.01 and 1.9

LogP threshold	Interaction effect	Interaction pval	Interaction logp
0.5	0.05	1.49E-05	4.83
1	0.06	1.87E-09	8.73
2	0.07	3.17E-12	11.50
3	0.07	8.14E-15	14.09
4	0.07	1.66E-13	12.78
5	0.07	5.98E-15	14.22
6	0.07	1.47E-13	12.83
7	0.07	4.51E-13	12.35
8	0.06	4.78E-12	11.32
9	0.07	4.78E-15	14.32
10	0.08	9.25E-17	16.03
11	0.08	7.70E-17	16.11
12	0.08	4.47E-17	16.35

Supplemental Table S4. Impact of logP fold threshold for including transcripts in an analysis of interaction between mutation and changes in transcript abundance, related to STAR Methods.

	Exc-DG		Exc-ET		Inh-MGE		Inh-CGE		ODC		Astrocytes	
	**Low**	**High**	**Low**	**High**	**Low**	**High**	**Low**	**High**	**Low**	**High**	**Low**	**High**
**No mutation**	1224434	115205	704927	63588	351714	36588	178097	24846	296262	29849	210020	32859
**Mutation**	19090	1962	10625	1026	5277	538	2027	261	3938	417	2142	261

Supplemental Table S5. The number of methylated sites with and without mutations in six cell types, related to Figure 5. The sites are shown for regions of low and high CpG density (where high is greater than 38 CpG/Kb)

	Exc-DG	Exc-ET	Inh-MGE	Inh-CGE	ODC	Astrocytes	Exc-CA3	Exc-CA1-Fras1	Exc-CA1-Camk2d	Exc-CA1-Galntl6
P- value	2.2E-25	3E-161	1.42E-49	3.63E-08	2.5E-74	0.386	3.38E-20	0.136	0.829	0.431
High density mean	0.031	0.113	0.083	0.041	0.09	0.011	0.036	0.184	0.032	0.062
Low density mean	-0.001	-0.005	-0.003	0.001	-0.021	-0.006	-0.004	0.005	0.072	0.008
t-value	10.412	27.125	14.822	5.509	18.284	-0.867	9.209	1.499	-0.217	0.789
df	134052	72084	42057	29678	34929	40890	71941	142	55	413

Supplemental Table S6. Results from t-tests to determine the difference between the number of sites with and without mutations in regions of high (>38 CpG/Kb) and low (<38 CpG/Kb) density CpGs in ten cell types, related to Figure 5.
